# Comparison of the dosimetries of 3-dimensions Radiotherapy (3D-RT) with linear accelerator and intensity modulated radiotherapy (IMRT) with helical tomotherapy in children irradiated for neuroblastoma

**DOI:** 10.1186/1756-6649-12-2

**Published:** 2012-06-28

**Authors:** Violaine Beneyton, Claudine Niederst, Céline Vigneron, Philippe Meyer, François Becmeur, Luc Marcellin, Patrick Lutz, Georges Noel

**Affiliations:** 1 Department of Radiation Oncology, Centre de lutte contre le Cancer Paul Strauss, BP42, 3, rue de la porte de l’hôpital, BP 62, F-67065, Strasbourg, cedex, France; 2 Pediatric Surgery Department, CHU Hautepierre, avenue Molière, F-67000, Strasbourg, France; 3 Pathology Department, CHU Hautepierre, avenue Molière, F-67000, Strasbourg, France; 4 Pediatric Oncology Department, CHU Hautepierre, avenue Molière, F-67000, Strasbourg, France

**Keywords:** Neuroblastoma, IMRT, 3D-RT, Tomotherapy, Dosimetry

## Abstract

**Background:**

Intensity modulated radiotherapy is an efficient radiotherapy technique to increase dose in target volumes and decrease irradiation dose in organs at risk. This last objective is mainly relevant in children. However, previous results suggested that IMRT could increase low dose, factor of risk for secondary radiation induced cancer. This study was performed to compare dose distributions with 3D-radiotherapy (3D-RT) and IMRT with tomotherapy (HT) in children with neuroblastoma. Seven children with neuroblastoma were irradiated. Treatment plans were calculated for 3D-RT, and for HT. For the volume of interest, the PTV-V_95%_ and conformity index were calculated. Dose constraints of all the organs at risk and integral dose were compared.

**Results:**

The conformity index was statistically better for HT than for 3D-RT. PTV-V_95%_ constraint was reached in 6 cases with HT compared to 2 cases with 3D-RT. For the ipsilateral kidney of the tumor, the V_12 Gy_ constraint was reached for 3 patients with both methods. The values were lower with HT than with 3D-RT in two cases and higher in one case. The threshold was not reached for one patient with either technique, but the value was lower with HT than with 3D-RT. For the contralateral kidney of the tumors, the V_12 Gy_ constraint was reached for all patients with both methods. The values were lower with HT than with 3D-RT in 5 of 7 children, equal in one patient and higher in one patient. The organ-at-risk volumes receiving low doses were significantly lower with 3D-RT but larger for the highest doses, compared to those irradiated with HT. The integral doses were not different.

**Conclusions:**

IMRT with HT allows a better conformity treatment, a more frequently acceptable PTV-V_95%_ than 3D-RT and, concomitantly, a better shielding of the kidneys. The integral doses are comparable between both techniques but consideration of differences in dose distribution between the two techniques, for the organs at risk, has to be taken in account when validating treatment.

## Background

The treatment of children with malignant tumors poses a great challenge to a pediatric oncology team. The standard management of neuroblastoma includes chemotherapy, surgery and radiotherapy. Irradiation doses are low, and a recent study from the Children’s Cancer Group showed a dose–response relationship with respect to local control, in which 20 Gy delivered to the primary site, had a better locoregional control rate than 10 Gy [1]. Children’s tissues are radiosensitive because they are still growing, and radiation treatment of neuroblastoma has been questioned. Because cancers in children are always chemosensitive, recent therapeutic protocols have attempted to decrease the use of radiotherapy according different ways [2-5]. Deleterious late effects can affect mainly musculoskeletal retardation due to the irradiation of the vertebrae and renal failure because of the proximity of the contrala-teral kidney and also of the ipsilateral kidney, if not removed with the tumor [6]. These late effects could have important consequences in terms of quality of life. Additionally, there is also the possibility of a second primary malignancy occurring in the field of irradiation or in other sites receiving small or large doses [7,8].

IMRT allows increased doses in a tumor volume in an attempt to improve disease control and allow decreasing doses in organs at risk to reduce complications or side effects and, consequently, to improve the quality of life. However, in pediatric treatment, IMRT raises some questions regarding dose distribution. Indeed, IMRT decreases the volume receiving the highest doses but increases the irradiated volumes at low doses because of the multiplication of fields creating a dose bath effect. Integral dose could allow integration of all of the dose scales and the generation of a global value of risk, but it remains to be evaluated clearly and fully.

In an attempt to provide a definitive answer, we proposed to theoretically compare the dosimetry used in seven children treated with irradiation for neuroblastoma. Dose distribution was calculated for 3D-radiotherapy delivered by linear accelerator (3R-RT) and for intensity modulated radiotherapy (IMRT) delivered by helical tomotherapy (HT).

## Methods

Seven children aged from 21 months to 5 years were included in this study. The disease was diagnosed because of abdominal pain in six cases and long-term fever in one case. Staging of all children was performed, including abdominal and lung CT scans, MIBG scans and catecholamine dosages, before treatment. At the end of the staging, all children had an International Neuroblastoma Staging System (INSS) score of 4 because of metastasis in the bone marrow for 5 cases, in the bones for 3 cases and in the abdominal nodes for 3 cases, with pleural effusion ac-counting for one case (Table 1).

**Table 1:** Children characteristics

#	Age	Gender	Localization	amplified Nmyc	State	Metastasis	Nephrectomy	Total removal	Site of irradiation	CTV-PTV margin	Total dose	Dose / fraction	Protocol of chemotherapy	Autograft of stemcell
1	2.5 yr	F	Right	Yes	IV	Abdominal nodes Medullar	Yes	Yes	Initial tumor site	5 mm	21 Gy	1.5 Gy	HNRBL1	yes
2	3 yr	F	Left	Yes	IV	Abdominal nodes Medullar Bone	Yes	Yes	Initial tumor site	5 mm	21 Gy	1.5 Gy	HNRBL1	Yes
3	5 yr	M	Left	Yes	IV	Pleural	No	Yes	Initial tumor site	5–10 mm	21 Gy	1.5 Gy	HNRBL1	Yes
4	3.5 yr	M	Left	No	IV	Bone	No	Yes	Initial tumor site	5–10 mm	21 Gy	1.5 Gy	HNRBL1	Yes
5	21 months	F	Left	No	IV	Medullar Bone	No	Yes	Initial tumor site T11-12 Sacrum	5–10 mm	21 Gy	1.5 Gy	HNRBL1	Yes
6	3 yr	M	Left	?	IV	Abdominal nodes Medullar	No	No	Initial tumor site	5–10 mm	21 Gy	1.5 Gy	HNRBL1	Yes
7	5 yr	M	Left	No	IV	Medullar	Yes	Yes	Initial tumor site	5–10 mm	21 Gy	1.5 Gy	HNRBL1	Yes

Chemotherapy was performed following the HNRBL1 protocol, which included different steps of the associated drugs (cisplatin, vincristine, carboplatin, etoposide and cyclophosphamide) given in a rapid delivery schedule [9,10] (Table 1). After chemotherapy, surgical resection was planned. Tumor removal included homolateral left nephrectomy in two cases and right nephrectomy in one case because partial nephrectomy was infeasible. Surgical resection was considered to be microscopically complete in 6 children and incomplete with probable viable tumor cells in the other case. After surgery, all patients were treated with high-dose chemotherapy, followed by autologous stem-cell transplantation. Five children were irradiated under complete anesthesia. Children were treated with tomotherapy. The protocol was approved by local ethics committee. Research was performed in compliance with the Helsinki declaration. Children’ parents signed consent after complete and clear information.

**Figure 1:**
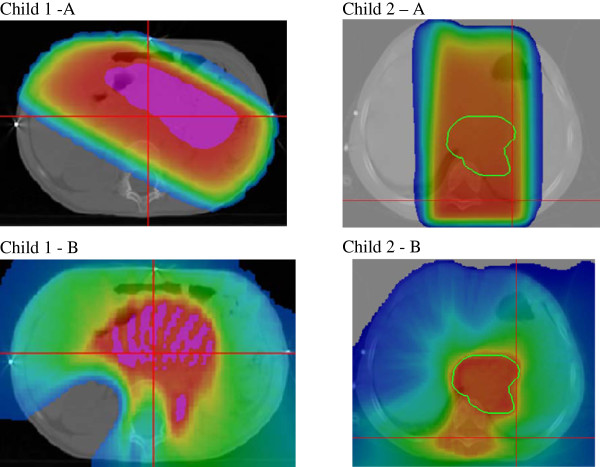
**Dose distribution 3D-RT (A) and HT (B) for children with oblique fields for RT-3D (child 1) and antero-posterior fields (child 2).** Red color: at least 95%, yellow: a least 90%; green at least 50% and blue at least 10% of the prescribed dose (~2 Gy). The 2-Gy isodose was shown because this dose is believed to be important for the secondary radiation-induced cancer risks.

All patients underwent CT simulation using a General Electric (GE) light-speed scanner (General Electric, Milwaukee, WI). Four-D CT was not assessed for these patients. The planning volume from the midthorax to the lower portion of the pelvis was scanned in 2.5-mm increments without contrast injection. Children were treated in the supine position in vacuum cradles for immobilization. The beam-on time varied from 142 to 412 seconds, relatively long compared with conventional techniques. The MV-CT imaging before treatment can also quite lengthy, requiring up to 10 minutes of imaging time per acquisition, fusion and correction of positioning. However, we previously controlled in the first children treated with tomotherapy the absence of intrafraction motion by a new positioning CT at the end of treatment. This question of beam-on time was not analyzed in our study and could be raise in concerns about intrafraction motion. Volumes were delineated with Focal (Computerized Medical System, St. Louis, MO, USA). The targeted volume was the initial tumor site that represented the CTV. This volume was delineated according to the initial diagnostic CT scan. The PTV was automatically delineated by adding a 5-10-mm 3D margin. The delineated organs at risk were the kidneys, vertebrae, liver, spinal cord, spleen, stomach and pelvic bone. The entire spinal cord was delineated. Dosimetries were calculated using Xio (Elekta AB, Stockholm, Sweden) and tomotherapy planning systems (Tomotherapy Incorporated, Madison, WI, USA). The dosimetric comparison was performed with Artiview (Aquilab, Lille, France). Radiotherapy was delivered at 21 Gy in 14 fractions for 5 days a week. The PTV was planned to receive at least 95% of the prescribed dose. Three-D-RT dosimetry was performed with two opposed, parallel antero-posterior or oblique-anterior and oblique-posterior fields, which were equally powered with 6 and/or 25 MV beams (Figure 1A). For IMRT with tomotherapy, the field width, pitch and modulation factors usually used for treatment planning and optimization were 2.5 cm, 0.287 and 2.5, respectively (Figure 1B). The dosi-metry was performed in regards of some organs at risk constraints. The volume of each kidney that received 12 Gy (V_12 Gy_) was limited to 20% in cases in which both kidneys were preserved and to <15% if only one kidney had been preserved [6]. Because of the risk of a lack of homogeneous vertebrae growth if a uniform dose was not delivered to this bone, a uniform dose into all the vertebrae proximal to the targeted volume was required, i.e., at least 80% of each irradiated vertebrae had to receive 80% of the prescribed dose. In all cases, vertebrae were included into CTV as they were in contact with or near the tumor. For the liver, no limit was proposed because a mean dose of 25 Gy into the total organ was considered acceptable [11] although some authors have advocated delivering a mean dose below 20 Gy when a combination of busulfan and melphalan is used as the protocol HNRBL1.

To attempt to obtain the best compromise we organized the constraints as following: V_12_ in the contralateral kidney, coverage of PTV, homogeneity in the vertebrae and V_12_ in the ipsilateral kidney.

### Statistics

For all organs, dose-volume histograms were calculated. For all organs at risk, the average doses were compared. Furthermore, we compared different factors for the target volume [12,13]: **PTV-V**_**95%**_ corresponds to the percentage of the PTV receiving at least 95% of the dose, according to the ICRU 50, and the **conformity index** (RIV/PTV) corresponded to the ratio between the reference isodose volume (RIV) and the PTV. We also calculated and compared the integral dose, which is the mean dose delivered to the total body minus the PTV (mean dose) / (noninvaded body volume—PTV). We also compared dose distributions in the organs at risk end for the total body.

Values were compared using the two-tailed nonparametric Wilcoxon test. Statistical analysis was performed using Statview 5.1 software (SAS industries).

## Results

### Targeted volume

**PTV-V**_**95%**_: The mean values were 95% (86.9–97.9) for HT and 88.7% (76.4–98.8) for 3D-RT. The difference in distribution was not statistically different (*p* = 0.3). This constraint was reached in 6 children on 7 with HT, compared to only 2 cases with 3D-RT (*p* < 0.05). In the child for whom the V95 constraint was not reached by HT, the value was 86.9%, as compared to 89.9% with 3D-RT (Tables 2 and 3)

**Conformity index**: The mean values were 1.57 (1–2.22) for HT and 2.93 (1.97–3.79) for 3D-RT. The difference in the distribution of the mean values was statistically significant (*p* = 0.02). The values were higher with 3D-RT in 6 cases, with a 3D-RT/HT mean ratio of 2.26 (1.37–3.79). In one case, the values were lower for 3D-RT than for HT, i.e., 1.97 and 2.1, respectively.

### Organs at risk

#### Irradiation of the homolateral kidney of the tumor or bed site

This topic concerned 4 children. The mean average dose was 6.8 Gy (3.4–14.3) for HT and 8.2 Gy (3.5–17.7) for 3D-RT. The V_12 Gy_ constraint was reached for 3 patients using both methods. The values were lower with HT than with 3D-RT in two cases and higher in one case but below the threshold. The threshold was not reached for one patient with both techniques, but the value was lower for HT than for 3D-RT. The mean V_12 Gy_ was 22.4% (4.6–57.8) for HT and 39.9% (15.4–100) for 3D-RT. The difference in the distribution was not statistically different (Tables 2 and 3).

#### Irradiation of the kidney contralateral to the tumor bed site

This measurement concerned 7 children. The mean average doses were 3.3 Gy (0.6–6.0) for HT and 2.2 Gy (0.5–3.4) for 3D-RT. The mean V_12 Gy_ was 2.3% (0–11.8) for HT and 2.8% (0–8.6) for 3D-RT. The V_12_ constraint was reached for all patients with both methods. The values were lower with HT than with 3D-RT in 5 of 7 children, equal for one patient (but 0% for child #4) and higher with HT than with 3R-RT in one patient (child #2), but the values were below the threshold. No differences in the distribution were statistically significant.

#### Irradiation of other organs at risk

The mean average doses are reported in Table 2. The differences in the distribution were not statistically significant with both techniques.

**Table 2:** Average mean value of the series

	**HT**		**3D-RT**	
	**Average mean value**	**Intervals**	**Average mean value**	**Intervals**
**Homolateral kidney**	6.8 Gy	3.4–14.3	8.2 Gy	3.5–17.7
**Controlateral kidney**	3.3 Gy	0.6–6.0	2.2 Gy	0.5–3.4
**Vertebrae**	11.5 Gy	4.4–18.3	11.3 Gy	5.4–18.6
**Sacroiliac joints**	4.7 Gy	0.03–20.8	3.6 Gy	0.00–20.6
**Liver**	6.2 Gy	4.6–9.8	5.4 Gy	1.5–10.6
**Spleen**	8.1 Gy	3.9–14.3	9.8 Gy	0.3–21.4
**Stomach**	11.0 Gy	6.5–15.5	12,2 Gy	3.8–21
**V**_**0.150 Gy/fraction**_	47.3%	38.8–65.1	32.2%	27.4–39.0
**Integral dose**	20.99 Gy/L	9.5–32.6	20.92 Gy/L	11.1–42.3
**V**_**95%**_	95%	86.9–97.9	88.7%	76.4–98.8
**Conformity index**	1.57	1.0–2.22	2.93	1.97–3.79

HT : helical tomotherapy ; 3D-RT : 3D radiotherapy.

### Dose distribution

**Figure 2:**
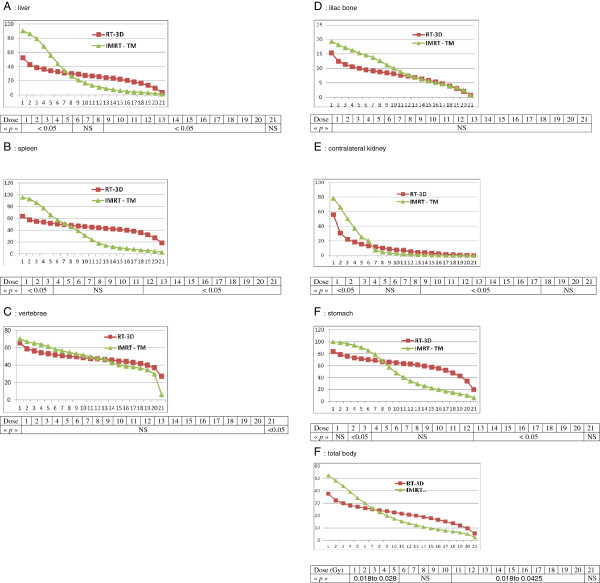
**Curves (IMRT TM—IMRT by tomothérapie and RT-3D) of mean organ or total body volumes irradiated at dose between 1 to 21 Gy.** Statistical signification dose by dose on table under curve.

The liver volumes receiving doses from 1 to 5 Gy were significantly lower for 3D-RT (*p* = 0.018). Between 6 and 8 Gy and at 21 Gy the volume distributions were not significantly different. Between 9 and 20 Gy, the liver volumes were significantly lower for HT (*p* = 0.0425–0.028). Curves intersect at a dose of 7 Gy. The spleen volume receiving doses from 1 to 3 Gy was significantly lower for 3D-RT (*p* = 0.0431–0.0464). Between 4 and 11 Gy, the volume distributions were not significantly different. Between 12 and 21 Gy, the spleen volumes were significantly lower for HT (*p* = 0.0425–0.028). Curves intersect at a dose of 8 Gy. The vertebrae volumes receiving doses from 1 to 20 Gy were not significantly different. At 21 Gy, the vertebrae volume was significantly lower for HT (*p* = 0.028). Curves intersect at a dose of 12 and 13 Gy. The pelvis bone volumes receiving doses from 1 to 21 Gy were not significantly different. The spleen volume receiving doses from 1 to 2 Gy was significantly lower for 3D-RT (*p* = 0.028–0.042). Between 3 and 8 Gy and between 18 and 21 Gy, the volume distributions were not significantly different. Between 9 and 17 Gy, the spleen volumes were significantly lower for HT (*p* = 0.0277–0.0464). Curves intersect at a dose of 6.5 Gy. The contralateral kidney volumes receiving doses from 1 to 3 Gy were significantly lower for 3D-RT (*p* = 0.0431–0.0464). Between 4 and 11 Gy, the volume distributions were not significantly different. Between 12 and 21 Gy, the spleen volumes were significantly lower for HT (*p* = 0.0425–0.028). Curves intersect at a dose of 8 Gy. The stomach volumes receiving doses from 2 and 3 Gy were significantly lower for 3D-RT (*p* = 0.0464). At 1 Gy, between 4 and 12 Gy and at 21 Gy, the volume distributions were not significantly different. Between 13 and 20 Gy, the spleen volumes were significantly lower for HT (*p* = 0.018–0.0425). Curves intersect at a dose of 8 Gy. The total body volumes receiving doses from 1 to 5 Gy were significantly lower for 3D-RT (*p* = 0.018–0.028). Between 6 and 9 Gy, the volume distributions were not significantly different. Between 10 and 20 Gy, the total body volumes were significantly lower for HT (*p* = 0.0425–0.018). Curves intersect at a dose of 7 Gy (Figure 2).

### Volume receiving a dose of 0.150 Gy per fraction

The mean percentages of the volume receiving less than 0.150 Gy per fraction were 47.3% (38.8–65.1) for HT and 32.2% (27.4–39.0) for 3D-RT. The percentage differences were statistically significant (*p* = 0.018) (Table 3).

**Table 3:** Patients’V_95%_, conformity index, V_12 Gy_ (%) for kidneys and integral doses according to helical tomotherapy and 3D-Radiotherapy dosimetry

	V_95%_		Conformityindex		Controlateral kidney V_12 Gy_		Homolateral kidney V_12 Gy_		Integral dose (Gy/mL)	
	HT	3D-RT	HT	3D-RT	HT	3D-RT	HT	3D-RT	HT	3D-RT
Patient 1	96.7%	94.5%	2.22	3.52	1.8%	7.6%	-	-	16.1	16.7
Patient 2	95.8%	81.0%	1.46	2.00	11.8%	0.1%	-	-	32.6	25.5
Patient 3	97.9%	76.4%	1.89	3.74	1.4%	2.4%	57.8%	100%	30.9	42.3
Patient 4	95.4%	97.1%	1.31	3.38	0%	0%	9.6%	20.8%	18.4	18.7
Patient 5	96.1%	83.3%	2.1	1.97	0.6%	1.0%	17.5%	15.4%	20.4	12.9
Patient 6	96.2%	98.8%	1.00	3.79	0.3%	8.6%	4.6%	23.3%	9.5	11.1
Patient 7	86.9%	89.9%	1.02	2.11	0%	0.2%	-	-	19.0	19.3

HT : helical tomotherapy ; 3D-RT : 3D radiotherapy.

### Integral dose

The mean integral doses were 20.99 Gy/L (9.5–32.6) for HT and 20.92 Gy/L (11.1–42.3) for 3D-RT. The difference in the distribution of the values was not statistically significant (*p* = 0.5) (Tables 2 and 3).

### In total—combination of constraints

If we consider the seven children who were relevant for the V_95%_ and contralateral kidney constraint, six (all except child #7) reached below-threshold values with HT, as compared to two children with 3D-RT (#4 and #6) (*p* < 0.05). If we consider the four children who were relevant for the V_95%_ contralateral and ipsilateral kidney constraints, 3 (#4–6) reached values below the constraints with HT, as compared to no children with 3D-RT (*p* < 0.05).

## Discussion

IMRT is an elegant technique for the treatment of children and allows a reduction of the high dose of radiation delivered to the tissues surrounding a tumor. To our knowledge, this article is the first publication to describe a dose comparison between tomotherapy and 3D-RT in neuroblastoma and is the third to compare IMRT and 3D-RT [14,15]. For pediatric irradiation, three goals have to be pursued: tumor control, avoidance of complications or sequellae and a decrease of the secondary radiation-induced cancer risk.

Because the contralateral kidney was close to the surgical bed and there was a need to deliver a homogeneous dose in the vertebrae, the PTV-V_95%_ constraint remains challenging with 3D-RT. In this series the PTV-V_95%_ constraint was achieved in 6 cases on 7 with HT compared to 2 cases only with 3D-RT. Furthermore, to obtain the best dose distribution with 3D-RT, the irradiated volumes are high, as demonstrated by the conformity index, which is approximately two-fold greater with 3D-RT compared to HT, (1.2 vs 2.9). Thus, HT achieved the first goal of pediatric irradiation, i.e., the possibility of better local control by improving the dose-to-tumor distribution. Conformity was already improved with other IMRT technique [15]. Although the risk of early or late complications at this dose is low, dose distribution to different organs have to be discussed, i.e., the kidneys, vertebrae. Because these young children also receive cisplatin, a well-known nephrotoxic agent, minimizing the radiotherapy dose to both kidneys is important because it may translate to less organ dysfunction. Differentiation has to be made between children with one or two remaining kidneys.

In cases in which the ipsilateral kidney had been removed, the shielding of the contralateral kidney must be strict. In our study, the overall values were very low and always below the dose thresholds for both radiotherapy techniques, and we did not observe statistically significant differences in the mean doses and V_12_ values between the two techniques. However, the V_12_ values were 18% lower for HT than for RT3D. In contrast, the average values were 50% higher for HT than for RT3D, i.e., 3.3 Gy and 2.2 Gy, respectively. This increasing of the dose with IMRT was described by Shaffer et al, but our figures were lower than those reported by these authors. The reason is probably that their children were treated with higher dose than in our series [15].

For the cases in which the homolateral kidney had been preserved, the main advantage of the IMRT could be its shielding of part of the remaining ipsilateral kidney, thus decreasing its risk of postirradiation involution. For the kidney ipsilateral to the tumor, the mean average dose was reduced by 17% with HT compared to 3D-RT, and the mean V_12 Gy_ was reduced by 43%. Some explanations for the lack of a more striking difference in shielding with HT include the following: (1) the need to irradiate vertebrae at higher doses than could be administered to avoid inhomogeneous growth of the bone and increase the dose to the contralateral kidney; and (2) technical reasons related to the tomotherapy system. To obtain a homogeneous dose in the target volume (PTV), the multileaf is opened to one size of the collimator width before and after the appearance of the target volume in the beam. This opening is total, i.e., not progressive. Thus, because we used 2.5 cm of collimator width, the size taken into account for irradiation was increased by 2.5 cm below and above the PTV in the cranio-caudal direction. Thus, compared to 3D-RT, the total beam’s cranio-caudal size was relatively higher with HT. Another point can be discussed is the motion of kidneys according to the respiratory control. A recent study concluded that the renal motion is highly correlated to age and weight of the child and diaphragmatic motion. However, authors concluded that, because of the low absolute magnitude of renal motion, the role of respiratory gating in younger children is limited [16].

Skeletal problems when using IMRT can be minimized by including adjacent vertebrae into the PTV. In a large series of children treated for Wilm’s tumor, Paulino et al differentiated children according delivered dose. RT dose was 1000–1200 cGy (Group A) in 12, 1201–2399 cGy (Group B) in 11, and 2400–4000 cGy (Group C) in 19. The 10- and 15-year actuarial incidences of scoliosis for Group A and B patients were 37.7 ± 12.4% and 37.7 ± 12.4%, whereas for Group C patients the incidences were 65.8 ± 12.0% and 74.4 ± 11.7% (*p* = 0.03) [17]. Although conventional 3D-RT delivers a somewhat homogeneous dose to the spine, HT with inclusion of the adjacent spine in the PTV offers the same homogeneous dose distribution, with no difference in mean doses. However, with the same dose distribution, the kidneys were better shielded, and the target volume, i.e., PTV, was better covered. Our conclusions are comparable with the previous comparison of 6 children published by Paulino et al [14] and cases analyzed by Shaffer et al; [15]. This is in accordance with Paulino et al study which advocated the inclusion of vertebrae in the CTV, in order to reduce dose heterogeneity and therefore reduce the incidence of skeletal growth deformities [14]. Even if Plowman et al attempt to demonstrate the contrary and advocate an increasing of integral dose by inclusion of vertebrae in the CTV, we cannot prove this assertion [18].

The goal of preventing radiation damage is crucial, particularly in children less than 3 year-old. However, radiation-induced carcinogenesis is not simply the result of mutations of stem cells. Several factors can confuse radiation causality, and sporadic cancer, genetic factors, lifestyle (including exogenous factors) and radiological irradiation are largely used for follow-up [8]. The role of the delivered dose is controversial. Proponents of a linear (no-threshold) relationship believe that the carcinogenic effect of any dose can be assessed by this relationship, while others claim that the role of low doses is underestimated because they do not take into account bystander effects. Conversely, radiobiologists and radiation oncologists have concluded that clinical experiences have not confirmed these allegations, and moreover that these low doses could induce an adaptive response in cells by stimulating the efficiency of DNA-repair capacities. In this study, body volumes receiving 1 to 5 Gy were significantly larger with HT than with 3D-RT. However, volumes receiving 7 and 10 Gy were equal between both techniques, and volumes receiving doses higher than 10 Gy were significantly smaller with HT than with 3D-RT. The difference of dose distribution is clearly related to the dose for all organs, with larger volumes irradiated at low dose with IMRT and larger volumes irradiated a high dose with RT-3D. The pathology type of the secondary tumors probably depends on the dose; The results already published based on this population demonstrated that the magnitude of the radiation dose received at the site of origin increased the risk of an second malignant cancer [19]. Thus, sarcoma occurs in the tissues receiving the highest dose [7,20]. Dose levels at which SMN are most likely to occur have not yet been clearly established. Kirova et al. [21] showed that most reported cases of radiation-induced sarcomas after breast irradiation occurred at sites that had received doses of 60–80 Gy, with a minimal dose of 10 Gy. Dörr and Herrmann [22] reported that the majority of second tumors occurred at sites that had received <6 Gy and were located within the margin region of the planning target volume (PTV), defined as the volume from 2.5 cm inside to 5 cm outside the margin of the PTV.

In a report on second malignancy in the United States, Wilms et al. showed that it is important to reduce the volumes receiving 20 Gy or more to decrease the risk of secondary cancers [23]. In our series, even if prescription doses were relatively low, i.e. 21 Gy, we show that HT should provide this opportunity by the decrease of the CI from 2.97 with 3D-RT to 1.57 with HT. Having demonstrated that the majority of second cancer had developed within volumes exposed to intermediate doses, Diallo et al suggest that tomotherapy and linear accelerator IMRT may increase the risk of second cancers by increasing the volume of intermediate dose regions. These results should be taken into account in the RT strategy [7].

It should be remembered that the issue of radiation-induced carcinogenesis is not without controversies. In particular, the phenomenon of radiation hormesis at low-radiation doses has attracted increasing attention [24]. Radiation hormesis is considered to be an adaptive response to the external stress of radiation exposure and is manifested in several cell lines in the form of reduced chromosomal aberrations and increased longevity. Extrapolating risk of radiation-induced carcinogenesis is an uncertain exercise. Data on radiation carcinogenesis are mainly derived from retrospective studies, with variable patient populations exposed to variable radiation doses whose dosimetry is often uncertain. In addition, a heightened risk of second malignancies may exist in these patients. In an extensive review of the literature, Suit et al*.* concluded that the experimentally observed heterogeneity in secondary induced-cancer risk indicates a large genetic role in determination of risk in the individual [20]. Furthermore, due to the quite large and undefined heterogeneity in the patient populations studied, no precise quantification of the risk of radiation-induced secondary cancer is available at present [20]. Most of the second cancer arising from irradiated volumes are not are not usually classified as radio-inducible cancers. Even if consequences in terms of second cancer are not yet a high-priority issue for radiation oncologists (comparing to the control of the cancer and the survival of patient), lowering the distant doses remains an important public health issue and a major challenge for RT in the future. A better understanding of dose distributions, inducible second cancer, for each organ, is necessary to perform dosimetry with real dose constraints to protect the development of second cancers. Also, prudence principle is required. In this goal, radiation oncologists are able to demonstrate some advantages of IMRT compared to 3D-RT.

## Conclusions

In conclusion, for children developing central neuroblastoma, IMRT with HT is a more efficient radiation technique than 3D-RT to ameliorate the dose coverage of the target volumes and to decrease the dose into the kidneys. Furthermore, the volumes receiving low doses are higher with HT, the volumes receiving the highest doses are lower with HT than with 3D-RT even if the integral doses were comparable between both techniques, and even if.

## Competing interests

Authors declare that they have no competing interest.

## Authors’ contributions

GN : conceived the study, performed the statistical analysis and wrote article. CN and PhM : carried out the dosimetry studies. VB: participated in the design of the study and analyzed data. All authors read and approved the final manuscript.

## Pre-publication history

The pre-publication history for this paper can be accessed here:

http://www.biomedcentral.com/1756-6649/12/2/prepub
